# Automated quantification of renal fibrosis with Sirius Red and polarization contrast microscopy

**DOI:** 10.14814/phy2.12088

**Published:** 2014-07-22

**Authors:** Jonathan M. Street, Ana Carolina P. Souza, Alejandro Alvarez‐Prats, Taro Horino, Xuzhen Hu, Peter S. T. Yuen, Robert A. Star

**Affiliations:** 1 Renal Diagnostics and Therapeutics Unit, National Institute of Diabetes and Digestive and Kidney Diseases, National Institutes of Health, Bethesda, Maryland

**Keywords:** Chronic kidney disease, fibrosis, Masson trichrome

## Abstract

**Abstract:**

Interstitial fibrosis is commonly measured by histology. The Masson trichrome stain is widely used, with semiquantitative scores subjectively assigned by trained operators. We have developed an objective technique combining Sirius Red staining, polarization contrast microscopy, and automated analysis. Repeated analysis of the same sections by the same operator (*r* = 0.99) or by different operators (*r* = 0.98) was highly consistent for Sirius Red, while Masson trichrome performed less consistently (*r* = 0.61 and 0.72, respectively). These techniques performed equally well when comparing sections from the left and right kidneys of mice. Poor correlation between Sirius Red and Masson trichrome may reflect different specificities, as enhanced birefringence with Sirius Red staining is specific for collagen type I and III fibrils. Combining whole‐section imaging and automated image analysis with Sirius Red/polarization contrast is a rapid, reproducible, and precise technique that is complementary to Masson trichrome. It also prevents biased selection of fields as fibrosis is measured on the entire kidney section. This new tool shall enhance our search for novel therapeutics and noninvasive biomarkers for fibrosis.

To listen to podcast click here

## Abstract (precis)

Histological assessment of interstitial fibrosis is commonly achieved by subjective scoring of Masson trichrome‐stained sections. We have developed an alternative approach that is rapid, reproducible, and precise by combining whole‐section imaging and automated image analysis with Sirius Red and polarization contrast microscopy. This new tool shall enhance our search for novel therapeutics and noninvasive biomarkers for fibrosis.

To listen to podcast click here

## Introduction

Interstitial fibrosis is a critical component for the progression of chronic kidney disease. Development of novel antifibrotic therapies and noninvasive imaging and liquid biomarkers capable of measuring progression would benefit from accurate histological measurement of fibrosis.

**Figure 1.**
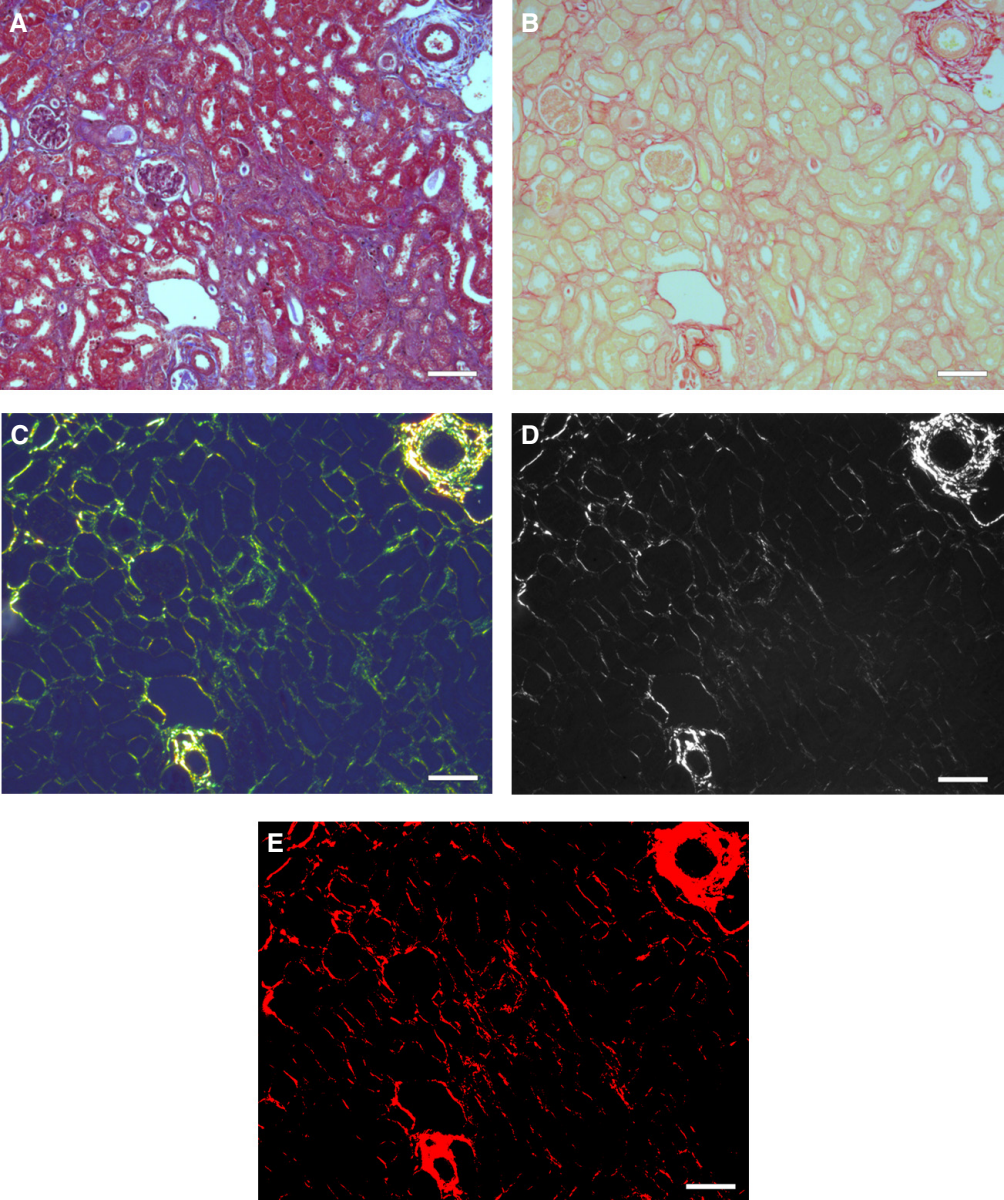
Kidney fibrosis. Color images of fibrotic kidney sections stained with (A) Masson trichrome (bright field) or stained with Sirius Red viewed under (B) bright field or (C) polarization contrast. Grayscale images of Sirius Red staining in polarization contrast (D) before and (E) after a background threshold is applied. Scale bar is 100 *μ*m.

Western blotting for collagen can accurately measure total kidney collagen, and hence fibrosis; however, it cannot discriminate between perivascular collagen and collagen from interstitial fibrosis. The Masson trichrome stain is widely used to measure tissue fibrosis. Fibrosis severity is assessed from an average of semiquantitative scores from 10+ “randomly” chosen fields. At best, ordinal scales are imperfect at assessing differences between observations, and attempts to more precisely quantify fibrosis with Masson trichrome have been further limited, because differentiating blue fibrosis from red background is challenging (Fig. 1A). Sirius Red is an increasingly used alternative, but the dark red stain can be equally difficult to interpret from light red background under bright field illumination (Fig. 1B). Binding of the Sirius Red molecule within the tertiary groove of collagen I and III fibrils enhances their natural birefringence. When viewed under polarization contrast, collagen appears bright against a dark background (Fig. 1C; Junqueira et al. 1978). We hypothesized that this stark contrast, when combined with computerized image analysis, would enable rapid and precise quantification of interstitial fibrosis. We compared our automated quantitative protocol with a previously established Masson trichrome semiquantitative protocol in a murine model of kidney fibrosis.

## Methods

### Animals and experimental design

We followed NIH criteria for the care and use of laboratory animals in research. Experiments were approved by the institutional ACUC. For the folic acid injury model 8‐week‐old male CD‐1 mice (Charles River Laboratories, Wilmington, MA) received 250 mg/kg folic acid i.p. (37.5 mg/mL/0.3 mol/L sodium bicarbonate), and kidneys were harvested at day 14. Blood urea nitrogen peaked at day 2 then fell back to approximately twice normal at day 14, as described previously (Doi et al. 2008). Mice with BUN above 100 mg/dL at day 2 were used for this study. For the unilateral ureteral obstruction (UUO) model 8‐week‐old male CD‐1 mice were anesthetized with isoflurane and then the ureter ligated immediately below the inferior kidney pole using 4–0 silk suture. Kidney tissue was harvested on day 10.

### Histology

Adjacent formalin‐fixed, paraffin‐embedded, 4‐*μ*m sections were stained with Masson trichrome or with Sirius Red (Puchtler et al. 1973; Junqueira et al. 1979). Masson trichrome staining was performed following the manufacturer's instructions (Accustain HT15, Sigma‐Aldrich, St. Louis, MO). Sirius Red staining was performed by incubating slides in 0.1% Sirius Red F3B for 1 h, washing twice in acidified water, dehydrating thrice in 100% ethanol, and then clearing in xylene.

### Microscopy

**Figure 2.**
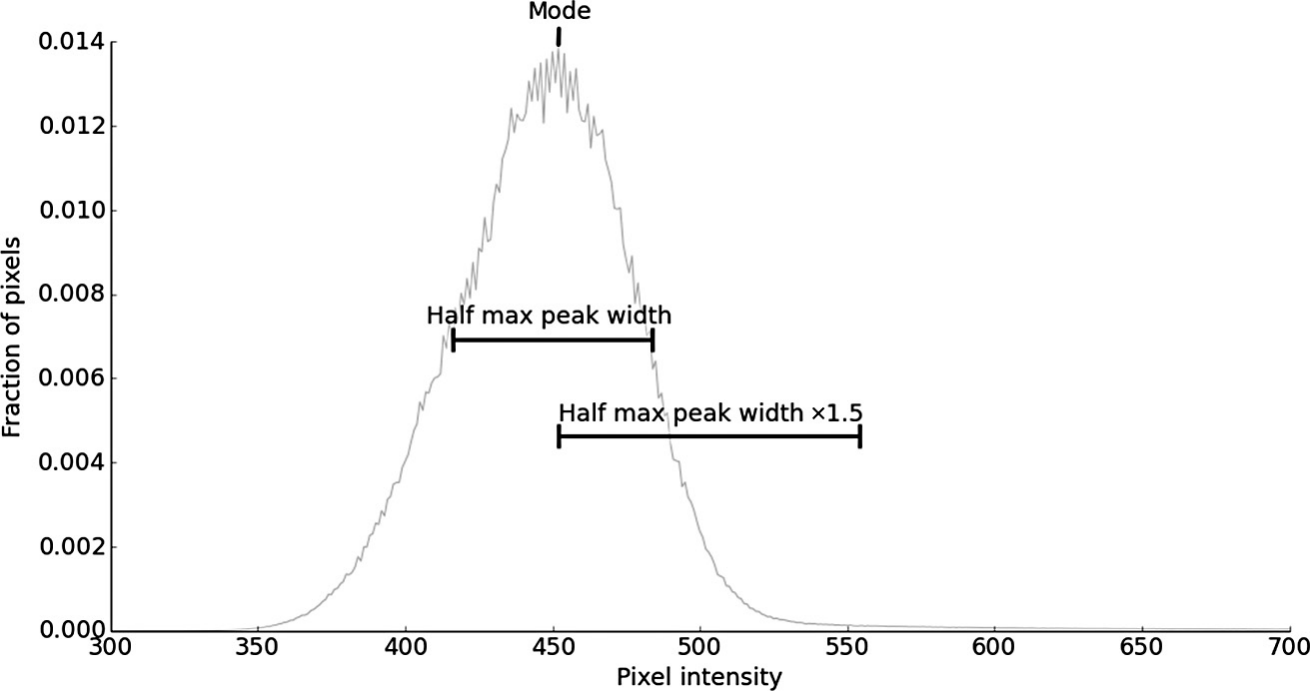
Threshold intensity is set by the distribution of pixel intensities in the image. Images of Sirius Red stained sections were recorded using 12‐bit resolution (possible pixel intensity values 0–4095). The location and width at half maximum of the background peak on a histogram are measured. The threshold is then set to the pixel intensity of the peak plus 1.5 times the peak width at half of the maximum intensity.

**Figure 3.**
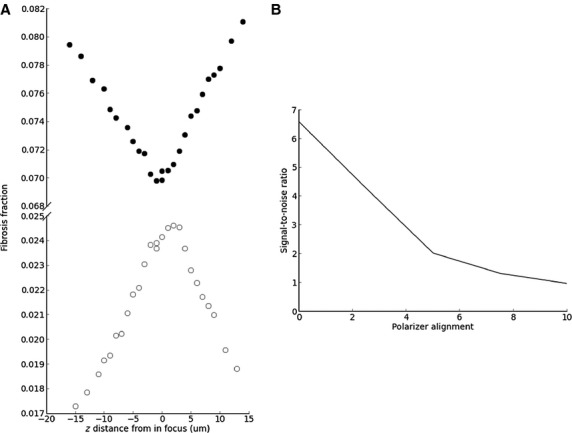
Focus and polarizer alignment influence the measurement of fibrosis. (A) A series of images both in focus and progressively out of focus were recorded for individual high‐power fields by varying the height of the stage. The percentage of fibrosis was measured for each image from high‐power fields with minimal fibrosis (open circles) or substantial fibrosis (closed circles). Loss of focus reduced fibrosis percentage area for fields with minimal fibrosis and increased fibrosis percentage area for fields with substantial fibrosis. (B) Signal‐to‐noise ratio for a HPF decreases as misalignment increases between the polarization filters.

Masson trichrome sections were viewed with bright field illumination at 20×. Ten nonoverlapping fields were scored with a semiquantitative ordinal scale (<5%; 5–10%; 10–25%; 25–50%; 50–75%; 75–100%) and the mean used as the fibrosis score. Sirius red sections were viewed with polarization contrast illumination. We kept lamp intensity, camera exposure, and camera gain constant, with special attention to orthogonal polarizing filter placement (uniform dark background aids background correction, Fig. 2) and image focus (focus nonlinearly alters fibrosis quantification, Fig. 3). Sirius red fibrosis was quantified (1) in 20 nonoverlapping 40× fields, and (2) over the entire kidney section using tiled 10× images.

### Image analysis to determine percentage fibrosis area

We wrote image analysis software (Jython scripts in Fiji (Schindelin et al. 2012); available from authors). The kidney was outlined manually to define the region of interest (ROI). We measured the percentage fibrosis within the ROI as follows: (1) the intensity distribution was used to set a background intensity threshold (Fig. 2). (2) Peri‐vascular regions were excluded from the ROI using a Gaussian filtering and thresholding technique. (3) Fibrosis was calculated as the percentage of unmasked pixels above threshold, relative to total pixels within the ROI.

**Figure 4.**
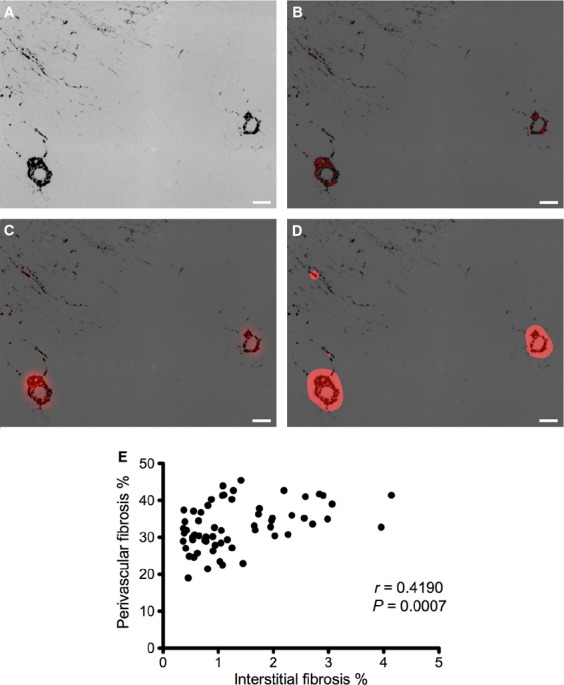
Masking perivascular collagen. Perivascular collagen was removed from the ROI using a mask. (A) Perivascular collagen appears as large clusters of high‐intensity pixels. (B) Applying a high threshold to the image preferentially identifies perivascular collagen. (C) A Gaussian blur assigns each pixel a weighted average of neighboring pixels. (D) Preservation of pixel intensity in large clusters enables a second threshold to identify perivascular collagen. Scale bar is 100 *μ*m. (E) Comparison of the perivascular and interstitial fibrosis percentage areas using the automated masking technique.

Perivascular fibrosis is easily identified from its localized, high‐intensity birefringence, and appearance (a large fraction of the pixels surrounding a dark hole are high intensity). In contrast, most interstitial fibrotic pixels were away from vessels, and of intermediate intensity, but with only rare, isolated, small clusters of high‐intensity pixels. In our algorithm, we applied a high threshold to isolate the high‐intensity pixels, then a Gaussian blur to assign each pixel a weighted average of neighboring pixels (Szeliski 2011). Away from vessels, preblur high‐intensity pixels in small clusters are predominately surrounded by low‐intensity pixels that (post blur) reduce the intensity of the cluster. Near vessels, pixels in large clusters are surrounded by high‐intensity neighbors so their weighted average remains high. Because low‐intensity pixels near a vessel are increased in intensity, the margins of large clusters expand and the lumens are filled in (Fig. 4). We then applied a threshold which excludes the small clusters yet includes the slightly expanded and filled large clusters representing the perivascular collagen staining.

## Results

**Figure 5.**
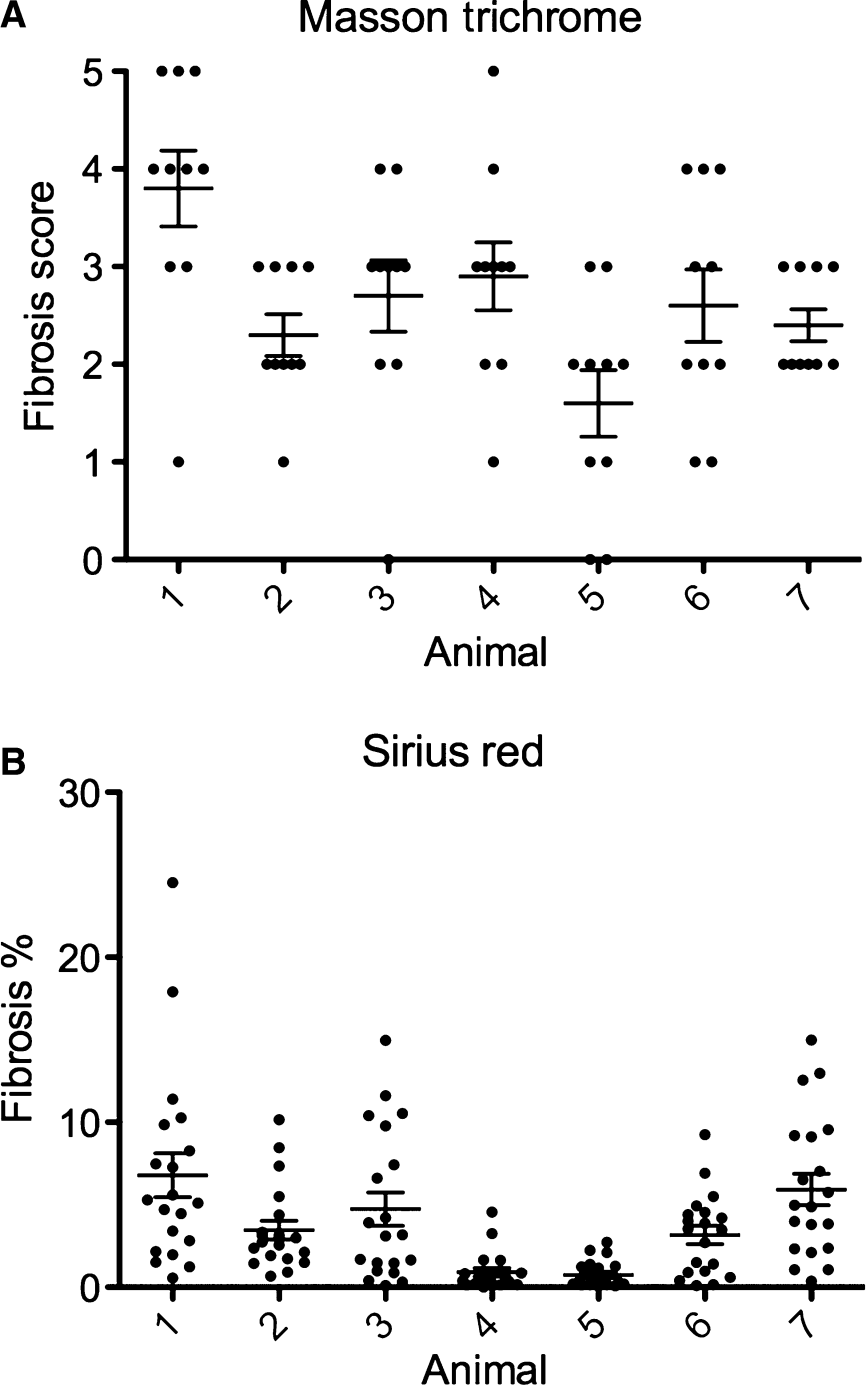
Severity of fibrosis varies among fields. Adjacent kidney sections from seven mice were stained with Masson trichrome or Sirius Red. Ten 20× objective fields from the Masson trichrome stained sections were scored for kidney fibrosis using a scale ranging from 0 to 5. Twenty 40× objective fields from Sirius Red stained sections were imaged using polarization contrast microscopy and the fibrotic area was measured. Horizontal lines represent the mean ± SEM. Both Masson trichrome fibrosis score (A) and Sirius Red percentage fibrotic area (B) varied between different high‐powered fields for the same animal.

Fourteen days after folic acid injection (see Methods), mice developed patchy renal fibrosis throughout the cortex and outer medulla adjacent to apparently normal tissue, with heterogeneity both within sections (Fig. 1) and between animals (Fig. 5). To assess the heterogeneity within a section, we measured kidney fibrosis in 10 or 20 random high‐power fields (hpf) on Masson trichrome‐stained or Sirius Red‐stained adjacent sections, respectively (Fig. 5). Individual field scores ranged from 10% to 75% with Masson trichrome, and 1–20% by Sirius Red. Values are consistently lower for Sirius Red (He et al. 2010), which measures the area of collagen fibrils, compared to Masson trichrome, which focuses on areas of affected tubules and cellular structures. Because of the extreme heterogeneity, we hypothesized that automated analysis of the entire section would enhance quantification of the overall level of fibrosis.

### Accuracy

**Figure 6.**
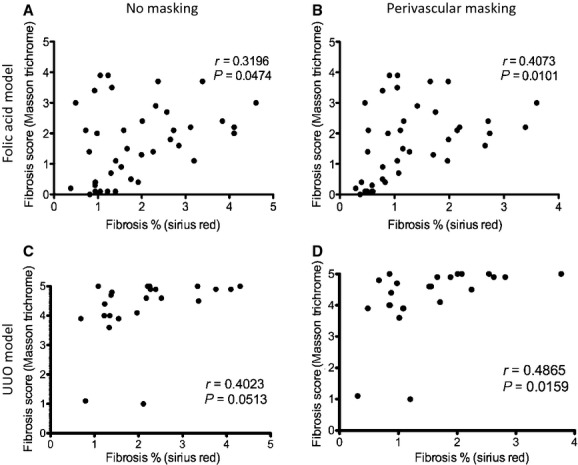
Correlation between fibrosis severity as measured by Masson trichrome and Sirius Red stains. For the folic acid model (A and B) and UUO model (C and D) adjacent kidney sections stained with Masson Trichrome or Sirius Red were scored and measured, respectively. The Masson Trichrome and Sirius Red fibrosis values were plotted, and a Pearson correlation coefficient calculated. (A and C) The entire region of interest was included when calculating the fibrotic area percentage using Sirius Red. Although statistically significant the relationship between Masson Trichrome score and Sirius Red fibrosis percentage was weak. (B and D) Vessels were excluded from the Sirius Red analysis by an automated three‐step threshold‐filter‐threshold process (Methods). The collagen surrounding many blood vessels is strongly birefringent and can erroneously increase the fibrosis percentage and weaken the correlation with the Masson Trichrome score.

We used the wide range of animal heterogeneity (Fig. 6) to test the agreement between the Sirius Red and widely used Masson trichrome methods. Individual Masson trichrome scores (average of 10 hpf) and Sirius Red areas (automated analysis of entire kidney section) correlated weakly, *r* = 0.32 (*P* = 0.047). We hypothesized that differences in how perivascular staining is scored (ignored by operators scoring Masson trichrome, but included by the automated Sirius Red method) contributed to this poor correlation. When we excluded Sirius Red high‐intensity perivascular areas (see Methods), the correlation improved to *r* = 0.41 (*P* = 0.010). The interstitial and perivascular areas of fibrosis were correlated, but there was a large range in perivascular fibrosis area values, particularly at low values of interstitial fibrosis (Fig. 4E), which may explain the improved agreement between Sirius Red and Masson trichrome after applying the perivascular mask. Hence, subsequent analyses were performed by automatically excluding perivascular areas.

Because different models may have different distributions of fibrosis, we investigated whether the agreement between Masson trichrome and Sirius Red is still robust in a UUO model. The Masson trichrome method considers 10 fields and the Sirius Red method considers the entire section. Due to this difference, the relationship between the methods may be altered for different distributions of fibrosis. Individual Masson trichrome scores correlated with Sirius Red fibrosis area percentages (*r* = 0.49, *P* = 0.0160) in the UUO model (Fig. 6D) suggesting that the correlation is robust to differences in fibrosis distribution.

### Reproducibility

**Figure 7.**
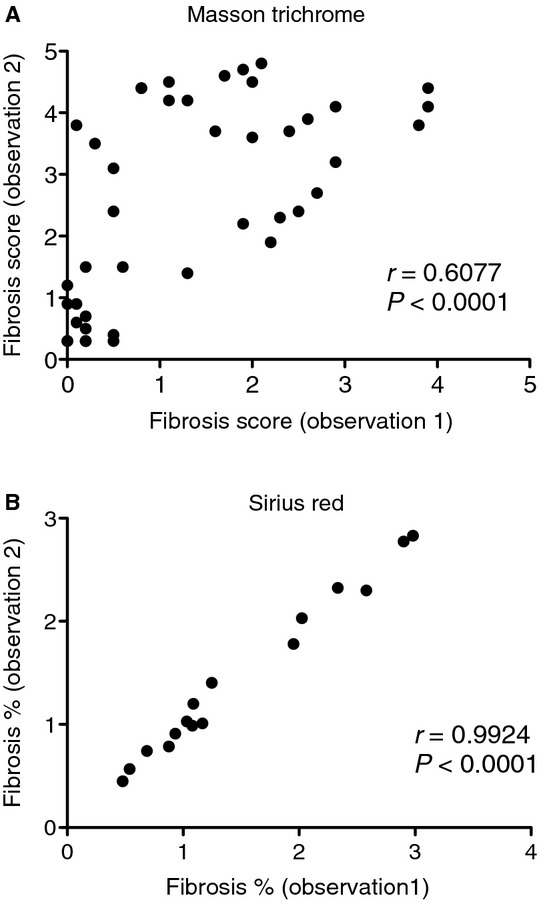
Correlation for Masson trichrome and Sirius Red stains between different sessions by a single observer. Two weeks after folic acid injection adjacent kidney sections were stained with Masson trichrome or Sirius Red. The sections were measured twice by the same observer for fibrosis. The correlation between the first and second observations for (A) Masson trichrome and (B) Sirius Red is plotted and a Pearson correlation coefficient calculated for each. Sirius Red was much more consistent between sessions than Masson trichrome, exhibiting a stronger correlation and less bias.

**Figure 8.**
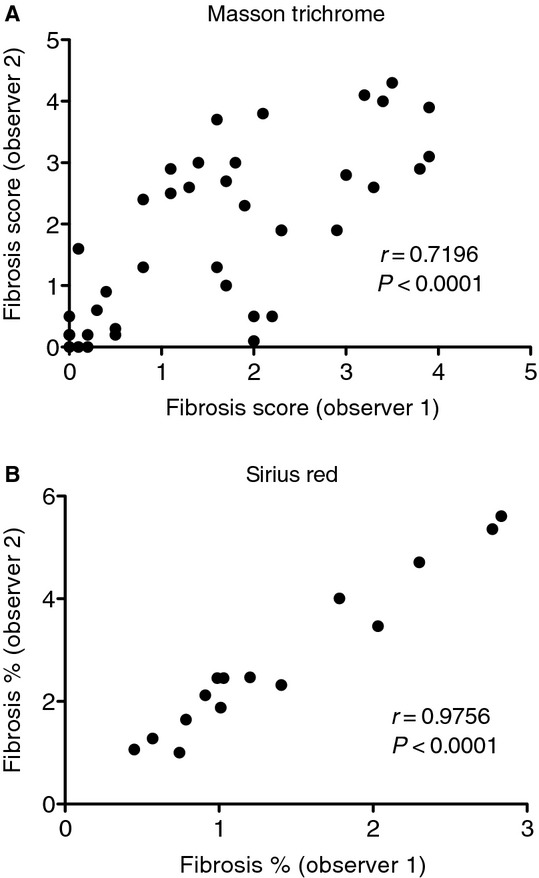
Correlation for Masson trichrome and Sirius Red stains between two observers. Two weeks after folic acid injection adjacent kidney sections were stained with Masson trichrome or Sirius Red. Two different observers then measured the sections for fibrosis. The correlation between the scores given by the two observers for (A) Masson trichrome and (B) Sirius Red is plotted and a Pearson correlation coefficient calculated for each. Sirius Red was more consistent between observers illustrating a reduced subjectivity of the automated three‐step threshold‐filter‐threshold process used with Sirius Red.

**Figure 9.**
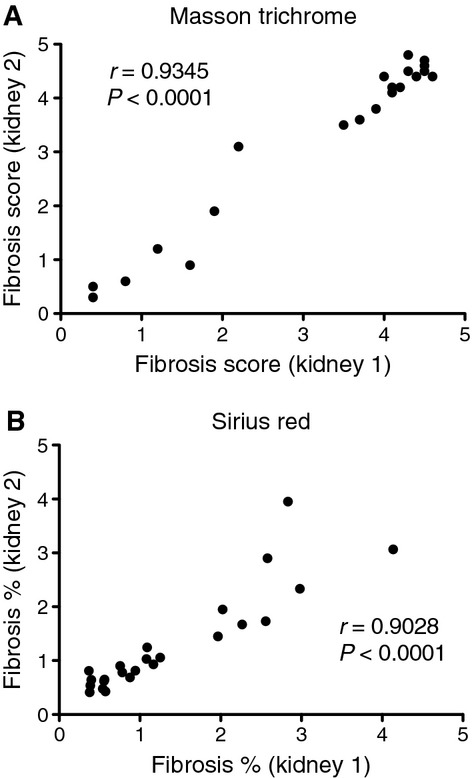
Correlation for Masson trichrome and Sirius Red stains between two kidneys from the same mouse. Two weeks after folic acid injection adjacent sections from the left and right kidneys stained with Masson trichrome or Sirius Red were measured for fibrosis. The correlation between the two kidney sections for (A) Masson trichrome and (B) Sirius Red is plotted and a Pearson correlation coefficient calculated for each. Although both techniques performed well, Masson trichrome scores were slightly more consistent between kidneys.

We next assessed reproducibility within sections, between operators, and between kidneys from the same animal for Sirius Red quantification, and Masson trichrome for comparison. First, we determined if repeating the same analysis on the same section gives similar results. For Masson trichrome and Sirius Red, the repeated sample (one operator) correlation coefficients were 0.61 and 0.99, respectively (Fig. 7). We next tested if the results are influenced by subjectivity from different operators. For Masson trichrome and Sirius Red, the correlation coefficients between operators were 0.72 and 0.98, respectively (Fig. 8). Introducing the additional variability of a second operator modestly lowered the correlation coefficient for Sirius Red, but increased that for Masson trichrome. We next compared the correlation of fibrosis between the right and left kidneys (*n* = 21–24). For Masson trichrome and Sirius Red, the correlation coefficients were 0.93 and 0.90, respectively (Fig. 9).

## Discussion

We have presented a method to measure interstitial fibrosis in the kidney. The most widely used method currently depends on manual scoring that is subjective and can limit reproducibility. Our automated Sirius Red/polarization contrast method for interstitial fibrosis uses whole‐section imaging and automated image analysis to achieve greater consistency than would be possible with a manual approach. The result is a high‐throughput, reproducible, and precise technique to quantify interstitial fibrosis.

### Background selection and vascular masking

Fibrotic signals were distinguished from background by a simple intensity threshold. We used the background peak mode plus 1.5 times the peak width at half height, which was the most reproducible among several methods tested (Fig. 2).

A variety of techniques have been used to measure renal fibrosis, each with its own advantages and disadvantages; therefore no single technique (“gold standard”) is – nor should be – universally used. Rather, multiple, complementary techniques are usually used to confirm the degree of changes in interstitial fibrosis. When compared, only modest agreements between techniques have been observed, at least in part due to differences in what is measured. For example, methods relying on homogenizing the kidney, such as hydroxyproline or western blot for collagen, sacrifice structural information (Woessner 1961; Farris and Colvin 2012). An advantage of histological techniques, such as Masson trichrome or Sirius Red, is that they preserve the architecture of the kidney, allowing anatomic location of the fibrosis to be ascertained. When perivascular collagen was included in our analysis, the Sirius Red fibrosis percentage area correlated poorly with Masson trichrome scores for interstitial fibrosis. An advantage of the current Masson trichrome technique is that it gives the operator an opportunity to exclude blood vessels when scoring. To approximate this process for the Sirius Red method, we developed an automated three‐step threshold‐filter‐threshold process that reliably identified perivascular regions without needing to manually locate each blood vessel. This automated masking improved the reproducibility and agreement with Masson trichrome.

### Differences between Masson trichrome and Sirius Red

Even after removing perivascular staining the correlation between Masson trichrome and Sirius Red was modest (*r* = 0.41). Although Sirius Red is increasingly used, few articles directly compare this technique with Masson trichrome, or any other technique (Whittaker et al. 1994; Hironaka et al. 2000; Hu et al. 2009; Farris and Colvin 2012; Huang et al. 2013). A low correlation was observed between Masson trichrome and Sirius Red in the heart (Whittaker et al. 1994) suggesting that these techniques may be fundamentally different. The difference observed may be due to: (1) each method considers different areas of the kidney. Masson trichrome scores the cortex and outer medulla, whereas Sirius Red considers the entire kidney. Excluding the inner medulla and inner stripe (~15% of kidney volume) from the Sirius Red analysis, matching the area scored for Masson trichrome, did not alter the correlation between the two methods (not shown). We measured the whole kidney section to minimize observer error. (2) The two methods use different scales. Masson trichrome uses a 6‐point scale (1–4 out of a maximum 5 observed) and Sirius Red reports a percentage value of the entire section (~0.5–5% observed). Potentially Sirius Red could take any value in the range 0–100% but as the interstitium is only a small fraction of the kidney cross‐sectional area, even a very fibrotic kidney would be expected to have a fibrosis area percentage that is only in the single digit range. (3) The two methods have different specificities. Sirius Red/polarization contrast is highly specific for type I and III collagen fibrils (Junqueira et al. 1978), whereas Masson trichrome stains a variety of matrix elements (Lillie 1940). Other groups have demonstrated greater correlation between collagen immunohistochemistry and Sirius Red than with Masson trichrome, although the correlation was low for both comparisons (Farris et al. 2011). We speculate that these stains detect different stages of fibrosis. Sirius Red stains higher ordered collagen fibrils found in more mature fibrosis, whereas Masson trichrome may also detect earlier fibrosis with ongoing inflammation (Grimm et al. 2003).

## Conclusion

Using whole‐section imaging in combination with automated image analysis, the Sirius Red polarization contrast method is rapid, reproducible, and precise. After a section is stained and scanned, fibrosis can be measured within 5 min. Sirius Red should not supplant Masson trichrome, but rather be viewed as a distinct stain with complementary properties. As the renal field is beginning to develop drugs that halt or even reverse fibrosis, this histological method adds to our armamentarium as an outcome measure to test the preclinical efficacy of drugs, noninvasive imaging methods and biomarkers.

## Conflict of Interest

None declared.
